# Discovering New Challenges in the Pharmacological and Non-Pharmacological Management of Insulin Resistance

**DOI:** 10.2174/0115733998379857251113060856

**Published:** 2026-01-15

**Authors:** Rina Amelia, Chandra Pranata, Tasia Dewi, Afra Meutia Nasution

**Affiliations:** 1 Department of Community Medicine/Public Health North Sumatra, Faculty of Medicine, Universitas Sumatera Utara, Medan, Indonesia;; 2 Faculty of Medicine, Universitas Sumatera Utara, Medan, Indonesia

**Keywords:** Diabetes mellitus, hyperglycemia, insulin resistance, lifestyle, physical exercise, herbal therapy

## Abstract

**Introduction:**

Insulin resistance has become a serious health problem over time. As people age, the incidence of insulin resistance increases. Genetic factors in families with a history of type 2 diabetes are also key risk factors. In Indonesia, an increase in the prevalence of type 2 diabetes to 8.5% was reported in 2018. This indicates that insulin resistance necessitates comprehensive management, encompassing both pharmacological and non-pharmacological approaches. This systematic review aims to discuss insulin resistance and its management, which can be achieved both pharmacologically through various available drugs and non-pharmacologically by modifying lifestyle habits that can be applied to patients with type 2 diabetes.

**Methods:**

Studies examining the comprehensive management of insulin resistance with antidiabetic pharmacological drugs and non-pharmaceutical interventions published between 2018 and 2023 were comprehensively searched using advanced searches on PubMed and Google Scholar, according to the specified inclusion and exclusion criteria.

**Results:**

Twenty studies were selected that met the inclusion criteria and have low risks of bias in most domains.

**Discussion:**

Non-pharmacological therapy, such as lifestyle changes that include a ketogenic diet, a vegetarian diet, a structured exercise intervention program, aerobic exercise, Continuous Positive Airway Pressure (CPAP), and electroacupuncture therapy, can significantly reduce insulin resistance and increase insulin sensitivity. Drugs currently prescribed for type 2 DM stimulate insulin secretion or increase insulin sensitivity.

**Conclusion:**

There are benefits of a plant-based diet, a ketogenic diet, and physical exercise in reducing insulin resistance non-pharmacologically. Meanwhile, pharmacologically, the first drug often given is metformin monotherapy. Combination therapy may be considered if monotherapy does not achieve the therapeutic target.

## 1 INTRODUCTION

Diabetes Mellitus (DM) is a metabolic disorder and multifactorial chronic health condition characterized by hyperglycemia, or abnormally elevated blood glucose levels, induced by various genetic and/or environmental factors [1-6]. Type 2 diabetes mellitus (T2DM), a form of DM, is a progressive chronic disorder characterized by hyperglycemia, resulting from either insulin resistance or elevated glucagon secretion, as described in studies [7, 8]. Typical manifestations of diabetes include polydipsia, polyuria, polyphagia, weariness, impaired eyesight, and weight loss [9]. Chronic hyperglycemia in people with T2DM results in prolonged damage and dysfunction of multiple organs, both acutely and chronically, particularly affecting the eyes, nerves, kidneys, blood vessels, and heart [10]. Chronic hyperglycemia leads to endothelial cell dysfunction and anomalies in hypoxia-induced neovascularization in the skin, nerves, heart, and skeletal muscle. This results from the ischemic response, which generates inadequate collateral blood vessels to compensate for the reduced neovascularization that often indicates an issue [11].

The World Health Organization (WHO) global report on diabetes indicates that the number of individuals with diabetes has nearly quadrupled since 1980, reaching 422 million [12], and is projected to rise to 693 million by 2045 [13]. This condition is characterized by elevated blood glucose levels resulting from insufficient insulin concentration and/or action, a pancreatic hormone that regulates blood glucose levels. The International Diabetes Federation (IDF) reports that 1 in 11 persons aged 20-79 years has T2DM, with a prevalence of 8.5% in the United States [7, 14]. RISKESDAS (Indonesian Basic Health Research) reported in 2018 that the prevalence of T2DM rose from 6.9% in 2013 to 8.5% in 2018 [15]. A nuclear family history of T2DM presents a risk three times greater than in individuals without such a history, particularly for those with both parents affected by the condition. In T2DM, there is a five to sixfold increase in the chance of children developing T2DM [16, 17]. Patients with T2DM exhibiting elevated mortality rates are predisposed to macrovascular complications, including stroke, accelerated atherosclerosis, and atrial fibrillation, due to the progression of cardiovascular disease. T2DM has become the primary cause of mortality and a significant risk factor for cardiovascular disease [18-24]. The mortality rate attributable to T2DM, as per global epidemiological data, exceeds 1 million fatalities annually [25].

T2DM frequently manifests in older individuals, those with obesity, a sedentary lifestyle, unhealthy diet, unhealthy sleep patterns, glucocorticoid medications, metabolic syndrome, and a familial predisposition to the condition [14, 26-36]. Individuals over 45 years of age, those who actively smoke, and those experiencing psychological stress and despair are additional risk factors for T2DM [37]. A study indicates that fifty percent of individuals with diabetes suffer from depression, primarily triggered by stress [38]. All psychiatric disorders, except dietary disorders, are associated with an elevated risk of developing T2DM [39-41]. The family can impact the patient's psychology, fostering adaptive coping mechanisms that contribute to an optimal psychological state and enhance quality of life [42].

Insulin resistance is a pathological state in which bodily cells fail to respond appropriately to insulin [43]. This results in elevated blood glucose levels and a rise in insulin secretion. This disorder involves the impairment of pancreatic beta cells, which secrete excessive insulin levels to manage hyperglycemia resulting from peripheral insulin resistance [44-46]. Insulin resistance may lead to consequences, including an increased risk of metabolic syndrome and cardiovascular disease [47]. Insulin resistance is projected to progress to type 2 diabetes mellitus (T2DM) within 10 to 15 years [48, 49].

Dyslipidemia is a primary risk factor for arteriosclerosis and cardiovascular diseases, characterized by abnormalities in the plasma lipid profile and reduced levels of high-density lipoprotein [50, 51]. Elevated blood glucose levels may damage both large and small blood vessels, leading to platelet aggregation that contributes to the development of atherosclerosis [52]. Dyslipidemia and T2DM are intricate conditions that involve adiposity-induced changes in β-cell function, adipose tissue biology, and multi-organ insulin resistance [53, 54].

The timely diagnosis could allow patients to implement the appropriate preventive and treatment strategies [55]. Diagnosis can be established using anamnesis, physical assessment, and corroborative investigations. In the medical history, both asymptomatic and typical symptoms may be present, specifically polyuria, polydipsia, polyphagia, and weight loss [56]. Physical examination may reveal problems associated with T2DM, including retinopathy, neuropathy, and cerebrovascular disease [57]. The primary laboratory assessments are the oral glucose tolerance test (OGTT) and hemoglobin A1c (HbA1c), which are used to evaluate therapeutic efficacy [58]. Some metabolite biomarkers that could be used to identify T2DM include alanine, glutamate, and palmitic acid [59].

The management of T2DM necessitates a comprehensive approach, incorporating both non-pharmacological and pharmacological therapies to prevent complications associated with the condition [56]. Measures that can be implemented include managing optimal body weight, regulating dietary intervention [60-66], engaging in physical activity, and taking oral hypoglycemic agents, such as those from the biguanide class, including metformin. Other medications that can be combined include those from the sulfonylurea, insulin, dipeptidyl peptidase-4 (DPP-4) inhibitor, sodium-glucose transport protein 2 (SGLT2) inhibitor, and glucagon-like peptide 1 (GLP-1) agonist classes [67, 68]. The primary medication used is metformin, which is deemed safe for individuals with an estimated glomerular filtration rate (eGFR) of ≥30 mL/min/1.73 m^2^ [69]. Physical activity constitutes one of the four essential components (education, food, physical activity, and medicine) in managing diabetes, which must be implemented to enhance the quality of life for diabetic patients. Vitamin D supplements and patient self-management of blood sugar levels may serve as alternatives for mitigating complications related to cardiovascular diseases in individuals with T2DM, thus reducing morbidity and death rates associated with the condition [70, 71]. If treatment with metformin and lifestyle modifications proves ineffective for three months, a combination of antidiabetic medications may be administered [72-74]. This systematic review will examine the holistic management of insulin resistance through various antidiabetic pharmacological agents and non-pharmacological interventions, including lifestyle modifications, surgical options, and sleep hygiene, anticipated to be utilized by clinicians to treat T2DM patients for improved outcomes.

## 2 MATERIALS AND METHODS

This systematic review was carried out by collecting, reviewing, and citing related articles. These articles were obtained through searches from search engines, such as Google Scholar, PubMed, Scopus, and Web of Science. These databases provided adequate coverage of the research question, offering a consistent search interface across multiple academic journals' databases. Keywords used in the search included “Insulin Resistance,” “Insulin Sensitivity,” “Treatment,” Pharmacology,” “Non-pharmacology,” and “Combination.” These keywords were combined using the Boolean search approach to make the search more restrictive and detailed. The selected articles were then reviewed based on the inclusion and exclusion criteria to ensure their relevance. The inclusion criteria included: 1) Non-Randomized Controlled Trials (NRCT), Randomized Controlled Trials (RCT), Systematic Review, and Meta-analysis articles, 2) Published in 2018-2023, and 3) Full-text articles in English. The exclusion criteria included 1) Articles other than discussions about insulin resistance and its pharmacological and non-pharmacological management, 2) Articles that only contain the abstract or partial parts of the full text, 3) Non-open-access articles or paid articles, 4) Duplication from other databases, and 5) Articles that acknowledge bias. Based on the results of this search, a systematic review was conducted across 20 journals. This study was conducted in accordance with the Preferred Reporting Items for Systematic Reviews and Meta-Analyses (PRISMA) 2020 guidelines for the reporting of research results. Multiple reviewers evaluated each article independently for methodological quality and strength of evidence to assess the risk of bias. Non-RCTs and narrative reviews were included when they provided context or supplemented limited data from RCTs. We ensured that discussions of study findings explicitly acknowledged these design differences to mitigate bias and enhance the transparency and reliability of our review outcomes.

## 3 RESULTS

Fig. (**1**) presents the PRISMA 2020 flow diagram, which illustrates the comprehensive process of article identification, screening, eligibility assessment, and final inclusion for this systematic review. Of the 36,344 articles initially traced, 8,044 were obtained from PubMed and 28,300 from Google Scholar. Then, 36,125 articles were excluded, 3,879 articles due to duplication, and 32,246 articles due to irrelevance. Two hundred nineteen articles were sought for retrieval, and 52 were not retrieved because they were non-open-access articles. Then, 167 articles were assessed for eligibility to be analyzed in detail, and 147 articles were excluded due to different outcomes (102 articles) and being written in a language other than English (45 articles). At the final stage, 20 articles were considered to meet the inclusion criteria.

Twenty articles deemed eligible in this literature review were with different study designs. Among the 20 articles, five were review articles, two were systematic reviews and meta-analyses, and 13 employed an RCT design. Out of the 13 articles with RCT study design included in this literature review, 1 article used a single-arm RCT, 1 adapted a three-arm RCT, 1 implemented a double-blind RCT, and the remaining 10 articles were RCT articles. The current study reviewed a total of 20 eligible articles to determine the non-pharmacological and pharmacological approaches to managing insulin resistance.

## 4 DISCUSSION

### 4.1 Non-Pharmacological Intervention

Table **1** presents several studies related to non-pharmacological research on BGL control in DMT2 patients. A lifestyle approach should be the primary non-pharmacological intervention for insulin resistance [75]. Non-pharmacological therapies, including lifestyle modifications, such as a ketogenic diet, a vegetarian diet, structured exercise intervention programs, aerobic exercise, and electroacupuncture therapy, can substantially reduce insulin resistance and improve insulin sensitivity. Exercise and lifestyle adjustments collectively mitigate the onset of insulin resistance. Continuous Positive Airway Pressure (CPAP) therapy has been shown to dramatically enhance glycemic control and reduce insulin resistance in patients with T2DM and obstructive sleep apnea (OSA) [76, 77].

#### 4.1.1 Dietary Modification

Medical nutrition therapy (MNT) is a non-pharmacological intervention applicable in the primary, secondary, and tertiary prevention of T2DM patients. MNT seeks to regulate blood glucose levels and is beneficial for comorbidities, including hypertension, dyslipidemia, and obesity. Calculations of carbohydrate calories are necessary for blood glucose regulation [78]. Moreover, plant-based diets can have a positive impact on both human health and the environment. It is recommended to increase the intake of fruits and vegetables rich in fiber, vitamin C, vitamin E, and magnesium, and low in saturated fat. This diet can mitigate the risk of T2DM, cardiovascular disease, hypertension, and cancer. This diet has several drawbacks, particularly the risk of deficiencies in protein, B vitamins, iron, zinc, and omega-3 fatty acids [79].

An RCT was undertaken, including fourteen overweight women diagnosed with polycystic ovarian syndrome (PCOS) who followed a Mediterranean ketogenic diet supplemented with phytoextracts (Kemephy) for a duration of 12 weeks. The outcomes of anthropometric and body composition assessments indicated a notable decrease in body weight (average weight loss of 9.43 kg; *p*<0,0001), body mass index (pre 28.84±2.10 *vs.* post 25.49±1.69; *p*<0.0001), blood glucose (pre 5.10±0.25 mmol/L *vs.* post 4.64±0.24 mmol/L; *p* < 0,0001), and insulin (pre 12.62±0.48 μU/mL *vs.* post 11.31±0.60 μU/mL; *p*<0.0001), accompanied by a considerable enhancement in Homeostatic Model Assessment of Insulin Resistance (HOMA-IR) (pre 2.85±0.15 *vs.* post 2.32±0.13; *p* < 0,0001) and lipid levels, such as triglycerides (pre 2.31±0.40 mmol/L *vs.* post 1.87±0.27 mmol/L; *p*<0.0008), total cholesterol (pre 5.36±0.36 mmol/L *vs.* post 4.72±0.33 mmol/L; *p*<0.0001), and low-density lipoprotein (LDL) (pre 3.11±0.60 mmol/L *vs.* post 2.33±0.17 mmol/L; *p*<0.0001), along with the rise of high-density lipoprotein (HDL) (pre 1.79±0.41 mmol/L *vs.* post 2.02±0.43 mmol/L; *p*<0.0001) [80].

The foods we consume significantly influence insulin resistance. This is particularly relevant for older adults and individuals with low physical activity levels. The increased consumption of processed and calorie-dense foods, including fast food, meat and meat products, processed cereals, and soft drinks, is believed to significantly contribute to the global rise in T2DM. A balanced and consistent diet can significantly mitigate insulin resistance and the likelihood of developing T2DM. It is important to enhance the intake of a healthy diet comprising foods with a low glycemic index, such as cereal fiber or a combination of whole grains and bran, as a dietary management strategy [10].

Dietary modifications should encompass alterations in eating habits, a decrease in energy consumption (to address excess body weight), and the avoidance of carbohydrates that excessively stimulate insulin production, specifically those with a high glycemic index and high glycemic load. This aligns with the hypothesis that food consumption stimulates glucose metabolism, resulting in heightened insulin synthesis by β cells and diminished glucagon secretion by α cells, thereby normalizing serum glucose levels. Post-secretion, insulin modulates blood glucose levels and promotes glucose storage as glycogen in the liver, muscle, and adipose tissue, leading to total weight gain [81].

#### 4.1.2 Physical Exercise

Physical activity is essential for enhancing insulin sensitivity, decreasing weight, mitigating the risk of cardiovascular disease, lowering blood cholesterol levels, reducing blood pressure, and improving overall health. The advised physical activity is moderate to high-intensity exercise for at least 150 minutes per week, spread over at least 3 days [79]. A systematic analysis was conducted using data from 846 individuals with T2DM and age-matched controls, comprising 440 participants in the intervention group and 406 in the control group. The average difference in fasting insulin levels was -1.64 (95% CI: -3.38 to 0.10), HOMA-IR 0.14 (-1.48 to 1.76), fasting blood glucose -5.12 (-7.78 to -2.045), HbA1c 0.063 (-0.82 to 2.08), and body mass index -0.36 (-1.51 to 0.79) relative to the control group. This suggests that therapies, such as regular exercise, improve insulin sensitivity in individuals with T2DM [82-84].

Physical exercise can enhance muscle insulin sensitivity and β-cell function in T2DM by promoting fatty acid oxidation and improving skeletal muscle mitochondrial activity. Additionally, it serves as a physiological stressor by activating glucose transporter type 4, facilitating glucose entry into the cell [10]. Recent evidence suggests that a combination of aerobic and weight training is more beneficial than either modality in isolation [84]. The findings of a study by Long *et al.* in 2022 further substantiate the assertion that a vegetarian diet, when paired with aerobic exercise intervention, exerts differential impacts on glycemic control and insulin resistance across diverse populations compared to pre-intervention, particularly regarding significant reductions in fasting blood glucose (SMD: − 0.27; *p*<0,001), HOMA-IR (WMD: − 0.75; *p*<0,001), and insulin levels (WMD: − 2.03; *p*=0,003) [79]. A vegetarian diet and an aerobic exercise regimen have been shown to enhance skeletal muscular strength, insulin sensitivity, and blood lipid profiles, facilitating weight loss and altering body composition.

The Framingham Heart Study demonstrated a substantial correlation between physical activity, sedentary behavior, insulin sensitivity, and blood levels of adipokines. Physical exercise was found to be correlated with increased insulin sensitivity, whereas sedentary behavior was positively linked to elevated levels of leptin and fatty acid-binding protein (FABP). Moreover, it has been noted that daily step count correlates positively with insulin-like growth factor-1 (IGF-1) levels and negatively with high-sensitivity C-reactive protein (hs-CRP) levels. In specific instances, incorporating pharmacological therapies is essential [83].

A systematic review conducted in 2023, encompassing 27 research articles, revealed an average difference of −0.75 (−1.08 to −0.42) for the homeostatic model assessment of insulin resistance, −0.27 (−0.30 to −0.23) for fasting plasma glucose, −1.10 (−5.06 to 2.86) for waist circumference, and −0.70 (−1.38 to −0.01) for body mass index. This indicated that participants who implemented a vegetarian diet alongside aerobic exercise exhibited markedly reduced fasting plasma glucose and insulin levels and improved body composition relative to their pre-intervention status [84].

A review of contemporary understanding of the significance of physical activity in diabetes care was conducted by Szlezak *et al.* in 2022. This review suggests that physical activity improves insulin sensitivity and is essential for maintaining glucose homeostasis. Physical activity has been demonstrated to enhance the body's glucose utilization, facilitating the maintenance of normoglycemia and improving lipid metabolism. It was found that individuals with T2DM frequently experience a range of chronic problems or comorbidities. Physical activity reduces the risk of developing T2DM through weight loss and improved insulin sensitivity. Consequently, with dietary modifications, an enhancement in physical activity should be instituted promptly in patients exhibiting glycaemic abnormalities, irrespective of the underlying etiology. However, these benefits may persist if the individual performs regular and consistent physical activity [85].

Research indicates that an appropriate diet and regular physical activity are far more efficacious in avoiding the onset of T2DM than any existing medication. Dudek A. and Otto-Buczkowska (2020) asserted that if the administered treatment yields unsatisfactory outcomes, it is imperative to augment the pharmacotherapy. The authors advocate for using incretin medications and SGLT2 inhibitors [86].

#### 4.1.3 Bariatric Surgery

Bariatric surgery is regarded as a useful intervention for preventing and managing T2DM in certain patients. Nonetheless, this approach presents numerous drawbacks, including potential shortages in iron, vitamin B12, folic acid, calcium, vitamin D, thiamin, protein, and fatty acids [80].

#### 4.1.4 Sleep Hygiene

Improving sleep hygiene is also crucial for regulating blood glucose levels. A sleep problem induces alterations in hormonal and metabolic functions, which can lead to the development of T2DM. Sleeping fewer than 6 hours daily increases the risk of T2DM [78].

#### 4.1.5 Acupuncture

Acupuncture is another non-pharmacological therapy that has been researched. An RCT conducted in 2022 involved female participants aged 18-40 years with PCOS and HOMA-IR, with 114 people randomly assigned to each group. Following four months of treatment, HOMA-IR exhibited alterations: a 14.7% reduction in the actual acupuncture group, a 25.0% reduction in the metformin group, and an 8.6% reduction in the sham acupuncture group. Genuine acupuncture showed inferior efficacy compared to metformin in enhancing HOMA-IR at four months post-initial consultation (difference, 0.6; 95% CI, 0.1-1.1). No notable variations were observed in HOMA-IR alterations between the authentic and sham acupuncture groups four months after the initial visit (difference, -0.2; 95% CI, -0.7 to 0.3). Despite being dropouts in this study, the dropout rates across different interventions were relatively similar. Specifically, 96 women (84.2%) in the true acupuncture group, 91 women (79.8%) in the metformin group, and 88 women (77.2%) in the sham acupuncture group completed all 48 sessions of treatment. This indicates no significant difference in adherence to the treatment protocols among the groups. Throughout the four-month therapy period, gastrointestinal adverse events were more prevalent in the metformin group, comprising diarrhea, nausea, anorexia, fatigue, vomiting, and abdominal discomfort (31.6%, 13.2%, 11.4%, 8.8%, 14.0%, and 8.8%). Bruising was more prevalent in the true acupuncture group (14.9%). This study revealed that genuine acupuncture did not enhance insulin sensitivity as effectively as metformin; however, it was superior to metformin in improving glucose metabolism, potentially reducing the incidence of T2DM, and exhibiting fewer side effects. Additional research is needed to evaluate the impact of acupuncture in combination with metformin on improving insulin sensitivity [82-88].

#### 4.1.6 Continuous Positive Airway Pressure (CPAP)

A meta-analysis examined the effects of continuous positive airway pressure (CPAP) therapy on glycemic regulation and insulin resistance in individuals with T2DM and OSA. The research encompassed data from three randomized trials with 202 participants. In comparison to the control group, CPAP treatment resulted in a notable decrease in insulin resistance (assessed by HOMA-IR), with a standardized mean difference (SMD) of -1.05 (95% CI: -1.91 to 0.19; p = 0.016) [76]. A comprehensive study and meta-analysis also investigated the efficacy of CPAP in preventing T2DM in patients with obstructive sleep apnea and insulin resistance. The results demonstrated that CPAP therapy enhanced the HOMA index [77]. In conclusion, CPAP therapy may successfully mitigate metabolic abnormalities linked to insulin resistance and obstructive sleep apnea as a non-pharmacological intervention.

While this systematic review identified several lifestyle interventions beneficial for managing insulin resistance, the comparative effectiveness of these interventions varies. CPAP therapy has been shown to significantly reduce insulin resistance in patients with T2DM and OSA, with a standardized mean difference (SMD) in HOMA-IR of -1.05, suggesting a substantial impact on glycemic regulation in this subgroup​. In contrast, the ketogenic diet, particularly when supplemented with phytoextracts as in the KEMEPHY protocol, demonstrated robust improvements across a broader metabolic profile, including significant reductions in body weight, fasting glucose, insulin, lipid levels, and HOMA-IR scores among overweight women with PCOS​. Although both interventions effectively enhance insulin sensitivity, the ketogenic diet offers broader metabolic benefits beyond glycemic control. However, CPAP may be especially critical for individuals with comorbid OSA, underscoring the importance of tailoring non-pharmacological interventions to patient-specific profiles and comorbid conditions.

### 4.2 Pharmacological Intervention

Insulin resistance is intricately linked to T2DM. Medications administered for T2DM can enhance insulin production or augment insulin sensitivity. Numerous T2DM drugs, including sulfonylureas, glucagon-like peptide 1 (GLP-1) agonists, and dipeptidyl peptidase-4 (DPP-4) inhibitors, focus on enhancing the insulin secretion capacity of β cells [88]. The management of patients with T2DM is complex, as there is now no curative medication available; nevertheless, glycemic levels can be regulated with pharmacological intervention. Furthermore, effective diabetes care encompasses accurate glucose control and the attainment of additional critical objectives, such as weight reduction, symptom alleviation, and the prevention of microvascular and macrovascular complications. A variety of antidiabetic medications produce therapeutic effects *via* distinct mechanisms. There are four primary categories of antidiabetic agents: (a) biguanides, exemplified by metformin, which diminish hepatic gluconeogenesis; (b) insulin secretagogues, which stimulate pancreatic insulin secretion and encompass sulfonylureas; (c) insulin sensitizers, which enhance peripheral tissue sensitivity to insulin, including thiazolidinediones; and (d) insulin itself (Table **1**) [89].

#### 4.2.1 Metformin

Metformin is the primary medication for insulin resistance. Metformin has received Food and Drug Administration (FDA) approval for treating T2DM and is also applicable for pre-diabetic individuals. Commonly reported side effects include abdominal pain, nausea, a metallic taste, bloating, and diarrhea. Metformin is the primary pharmacological treatment for T2DM. This medicine reduces glucose levels and enhances insulin sensitivity, affecting various organs, including the liver, skeletal muscle, endothelium, adipose tissue, and ovaries. Only if the HbA1c level exceeds 7.0% after three months can additional therapy be implemented. In a previous study comparing metformin monotherapy to other agents, metformin induced a decrease in HbA1c of 1-1.8%, which was more effective than DPP4i [87]. Regrettably, metformin exhibits numerous adverse effects, ranging from mild to severe, which contribute to suboptimal adherence, rendering it the oral antidiabetic treatment with the lowest compliance rates [90]. Moreover, thiazolidinediones (TZDs) and metformin are insulin-sensitizing antidiabetic agents that address adipose tissue fat storage and hepatic glucose production.

Although numerous diabetes medications are available, monotherapy with alternative drugs has demonstrated ineffectiveness in adequately managing blood glucose levels and associated comorbidities; consequently, therapeutic management frequently necessitates combination therapy utilizing agents with diverse mechanisms of action (Fig. **2**). Fig. (**2**) depicts the classification of glucose-lowering medications used in the management of insulin resistance and T2DM. It categorizes these medications by their mechanisms of action and includes commonly used drug classes, such as biguanides (metformin), GLP-1 receptor agonists, DPP-4 inhibitors, SGLT2 inhibitors, and insulin [74].

This strategy may be influenced by issues related to polypharmacological methods, including side effects, toxicity, and adverse drug-drug interactions. Nonetheless, the advantages of these combinational medicines are frequently undermined by inadequate patient adherence, which could be enhanced by formulating two or more active ingredients in a single tablet. A review of studies by Taylor *et al.* in 2021 advocates for metformin as the primary treatment to reduce HbA1c in T2DM and identifies four additional pharmacological options for patients failing to achieve therapeutic goals: DPP-4 inhibitors, GLP-1 agonists, SGLT2 inhibitors, or TZDs, aligning with the guidelines set forth by the American Diabetes Association (ADA) and the European Association for the Study of Diabetes (EASD) in 2020 [74, 83]. Comparable findings were also reported in a 2019 review of research by Tagi *et al.*, indicating that metformin is the primary medication for insulin resistance in children. In addition to providing GLP-1 agonists, DPP-4 inhibitors, SGLT1, and SGLT2 inhibitors may be utilized as pharmacological treatments for insulin resistance in children [90].

Furthermore, the findings of a 2022 study analysis by Reifsnyder *et al.* elucidated that metformin administration did not influence insulin tolerance or HOMA-IR [92]. Metformin is also associated with adverse effects, including nausea, vomiting, and abdominal discomfort [91]. Nevertheless, the combination of metformin with rapamycin significantly elevated HOMA-IR while mitigating the negative effects of either drug alone. Specifically, metformin prevented rapamycin-induced hyperglycemia and islet degranulation, while rapamycin blocked the nephrotoxic influence of metformin [92].

#### 4.2.2 GLP-1-Agonist, DPP4i, and SGLT-2 inhibitors

Glucagon-like peptide-1 agonists function by a mechanism involving synthetic glucagon-like peptide-1, a digestive hormone that diminishes inflammatory cytokines in adipose tissue, the liver, and vascular walls. DPP4 inhibitors are novel oral hypoglycemic agents that diminish incretin inhibition, stimulating insulin production. SGLT1 is the primary transporter for glucose and galactose absorption during digestion, while SGLT2 functions in the kidneys. SGLT-2 inhibitors are among the endorsed oral antidiabetic medications. The mode of action of this OAD involves the inhibition of the transporter. Inhibition of this transporter demonstrates enhanced glycemic regulation and decreased HbA1c levels.

Table **1** summarizes key statistical outcomes from the reviewed studies.

### 4.3 Combination of Pharmacological and Non-Pharmacological Intervention

Non-pharmacological interventions, including lifestyle changes, can be extremely effective in improving metabolic control, lowering the risk of diabetes, delaying its progression, and enhancing overall quality of life in the long term. However, pharmacological intervention yields greater short-term effects. Metformin has been demonstrated to reduce insulin resistance by decreasing fasting plasma glucose and insulin concentration in earlier RCTs. Furthermore, a previous 5-week double-blind RCT on 21 obese adolescents reported that GLP-1 analogs could improve body weight, fasting plasma glucose, HbA1c, and fasting serum insulin levels. However, none of these effects were statistically significant, possibly due to short trial duration and a small number of participants (Table **1**) [83].

Nonetheless, pharmacological intervention is often initiated in conjunction with behavioral modification to achieve the best outcomes. In addition, insulin action and glycemic control improvements are regularly observed with caloric restriction and reduced adipose mass, whether accomplished by diets, bariatric surgery, SGLT2 inhibitors, or GLP-1 RA [88].

The research conducted by Rahman *et al.* also supports the use of metformin, when combined with electroacupuncture therapy, to increase insulin sensitivity. This is based on the theory that electroacupuncture increases insulin sensitivity through the regulation of Sirtuin-1 and peroxisome proliferator-activated receptor-γ coactivator-1α [36]. Electroacupuncture can also increase peripheral blood flow and glucose uptake in skeletal muscle in response to muscle twitch reflexes during manual or electrical stimulation. This research demonstrates a relationship between electroacupuncture and increased insulin sensitivity [93].

In contrast to the findings of a study by Wen *et al.* (2022), it was determined that acupuncture did not enhance insulin sensitivity as effectively as metformin and exhibited a greater frequency of gastrointestinal side effects compared to the acupuncture group, aligning with the results of a study review by Tagi *et al.* [82, 83]. However, this study has several limitations, including a relatively small sample size, and participants who withdrew due to pregnancy did not undergo further blood tests, which could introduce bias.

The research conducted by Rahman *et al.* also had limitations, namely that the diet and exercise patterns of the research participants were not closely monitored, which introduced the possibility of bias. Furthermore, the duration allocated for this research was limited, preventing researchers from conducting long-term observations. However, research conducted by Rahman *et al.* is the first study in Indonesia to investigate the effectiveness of a combination of electroacupuncture therapy and pharmacological treatment for PCOS in Indonesia [81].

Contrary to findings from other studies, a 2021 review by Lee *et al.* supports the notion that therapies aimed at reducing hepatic diacylglycerol (DAG) and acetyl-CoA levels by inhibiting hepatic de novo lipogenesis may effectively address hepatic insulin resistance in T2DM [94].

Insulin resistance can also increase a patient's susceptibility to atherosclerosis and cause disturbances in platelet function in blood vessels, resulting in an increased risk of cardiovascular complications [95, 96]. To prevent and reduce this risk, the use of statins and antiplatelet agents as secondary prevention for cardiovascular complications in T2DM patients is recommended [97, 98]. However, some doctors are reluctant to prescribe statins, especially in individuals at risk of developing diabetes. This refers to a meta-analysis conducted by Sattar *et al.* in 2010, which is often cited as showing a 9% increase in the incidence of diabetes in individuals taking statins [99].

Therefore, in 2023, a meta-analysis-based study conducted by Jimenez *et al.* updated the evidence on the diabetogenic effect of statins and found that statins increase the risk of T2DM [92, 100]. However, this meta-analysis has limitations. A total of 39% of studies that met the inclusion criteria failed to show a significant difference between statins and their placebo controls.

## 5 LIMITATIONS

This systematic review faced multiple constraints in its evidentiary foundation and methodologies. Numerous research studies included relatively small sample sizes, which constrain the generalizability of the results. The absence of consistency in techniques, particularly in intervention procedures and outcome measurements, hinders direct comparisons among studies. Certain NRCTs and RCTs exhibited potential biases due to limited participant follow-ups and inadequate management of confounding variables, including nutrition and exercise habits. The review's dependence on solely English-language articles and its disregard for non-open-access studies may introduce selection bias. This restriction was primarily due to feasibility constraints and the need to ensure transparency and accessibility of full-text articles for thorough analysis. As a result, relevant studies published in other languages or behind paywalls may have been excluded, potentially limiting the diversity of perspectives and the comprehensiveness of the evidence base. We acknowledge that this could lead to the underrepresentation of data from non-English-speaking regions, particularly studies from low- and middle-income countries (LMICs), which may have distinct contextual insights. Future reviews should consider multilingual search strategies and collaborations with international researchers to capture a broader and more representative range of studies. The brief duration of numerous therapies limits the comprehension of long-term consequences, a vital consideration in managing chronic illnesses, such as insulin resistance.

The generalizability of this study's findings may be limited by the predominantly small and geographically concentrated study samples. Cultural, dietary, and genetic differences can significantly influence responses to both pharmacological and lifestyle interventions. For example, variations in diet, physical activity norms, and genetic predispositions may affect insulin sensitivity and treatment outcomes. These factors underscore the need for more diverse, multiethnic research to ensure broader applicability of the findings.

The findings emphasize the necessity of combining pharmaceutical and non-pharmacological strategies to manage insulin resistance. Healthcare practitioners should prioritize personalized treatment approaches that incorporate lifestyle adjustments, plant-based diets, and structured exercise regimens, along with pharmaceutical interventions, such as metformin. Policymakers should advocate for public health programs that enhance understanding of lifestyle factors leading to insulin resistance and facilitate access to affordable therapies. The findings of this review also underscore the urgent need to integrate pharmacological and non-pharmacological interventions into national diabetes management guidelines, particularly in LMICs, where the burden of insulin resistance and T2DM is rising rapidly. Implementation strategies should emphasize the accessibility and affordability of first-line therapies, such as metformin, while promoting culturally adapted lifestyle interventions, including plant-based or low-glycemic diets and structured exercise programs. Public health frameworks should support task-shifting models, enabling trained community health workers to deliver education and behavioral interventions. Scalable solutions, such as mobile health platforms, also facilitate patient monitoring and adherence in resource-constrained settings. Integrating these approaches into national clinical practice guidelines would standardize care and enhance health system responsiveness to the growing diabetes epidemic in LMICs.

Additionally, to strengthen the evidence base and enhance clinical decision-making, future research should focus on well-designed, multicenter RCTs that directly compare the efficacy of non-pharmacological and pharmacological interventions in specific populations. For instance, RCTs comparing CPAP therapy with electroacupuncture in patients with T2DM and comorbid obstructive sleep apnea could elucidate which modality offers superior improvements in insulin sensitivity and glycemic control. Similarly, head-to- head trials assessing metformin against newer agents, such as SGLT2 inhibitors, in subgroups like obese individuals with early-stage insulin resistance or women with PCOS would provide valuable insights for personalized treatment strategies. Future studies should prioritize long-term follow-up to assess the sustainability of outcomes and consider culturally diverse cohorts to improve generalizability across global populations.

## CONCLUSION

This current systematic review of twenty articles found that there are benefits of plant-based diets, ketogenic diets, physical exercises, and CPAP therapy in reducing insulin resistance non-pharmacologically. Meanwhile, pharmacological interventions were found to directly target specific physiological pathways to improve glycemic control. The first-line drug that is often given is metformin monotherapy. A second drug may be considered if monotherapy does not achieve therapeutic targets. If the patient does not reach the therapeutic target with metformin plus DPP4 inhibitor, then metformin plus GLP-1 receptor agonist can be considered. Furthermore, if administered simultaneously, the rapamycin/metformin combination may synergize in reducing insulin resistance while mitigating adverse events. Hence, combining non-pharmacological and pharmacological management, such as the electroacupuncture/metformin combination, may offer comprehensive and sustainable benefits in increasing insulin sensitivity.

## AUTHORS’ CONTRIBUTIONS

The authors confirm their contribution to the paper as follows: study conception and design: RA; data collection: CP and TC; data analysis or interpretation: AM; methodology: RA and CP; investigation: AM; draft manuscript: RA and CP. All authors reviewed the results and approved the final version of the manuscript.

## Figures and Tables

**Fig. (1):**
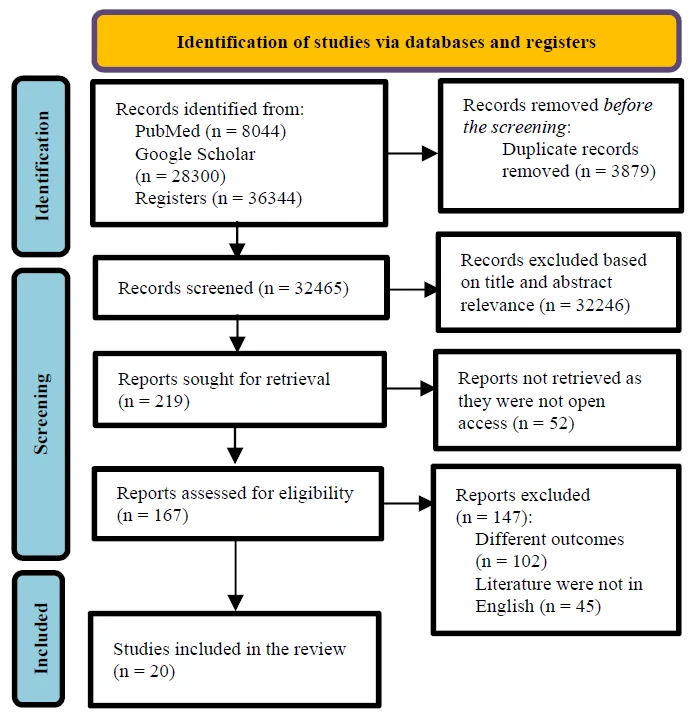
PRISMA 2020 flow diagram illustrating the study screening, selection process, and reasons for exclusion of studies.

**Fig. (2):**
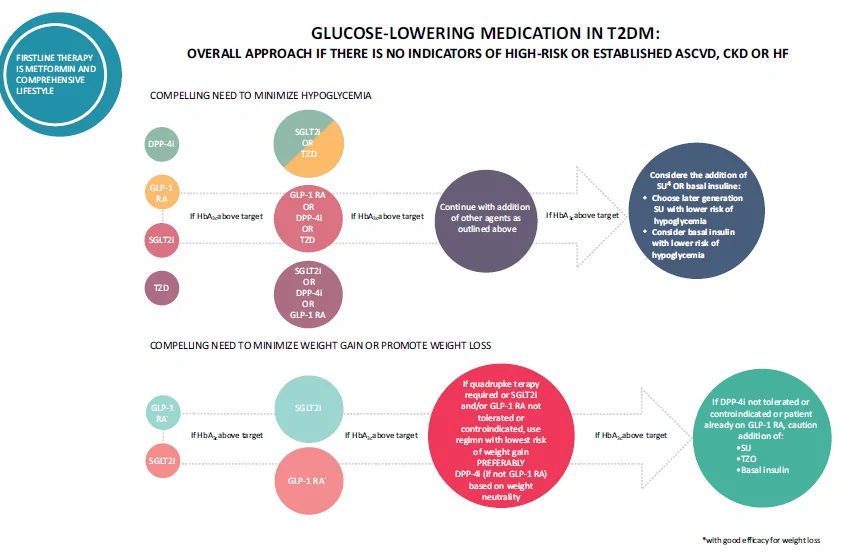
Glucose-lowering medication. **Abbreviations:** T2DM: type 2 diabetes mellitus; ASCVD: atherosclerotic cardiovascular disease; CKD: chronic kidney disease; HF: heart failure; DPP-4i: dipeptidyl peptidase-4 inhibitor; GLP-1 RA: glucagon-like peptide-1 receptor agonist; SGLT2i: sodium-glucose cotransporter-2 inhibitor; TZD: thiazolidinedione; HbA1c: hemoglobin A1c; SU: sulfonylureas.

**Table 1:** Key statistical outcomes from the reviewed studies.

**Title of the Study**	**Intervention**	**Sample Size (N)**	**Outcome**	**Effect Size**	**95% CI**	***p*-value**
Early Detection of Macrovascular Complications in Type 2 Diabetes Mellitus in Medan, North Sumatra, Indonesia: A Cross-Sectional Study [18]	Exercise and GLUT-4	RCT	HbA1c and glucose levels	Improved	Not reported	<0.05
Ketogenic Diet Benefits to Weight Loss, Glycemic Control, and Lipid Profiles in Overweight Patients with Type 2 Diabetes Mellitus: A Meta-Analysis of Randomized Controlled Trials [60].	Ketogenic diet in T2DM	Meta-analysis	Glycemic control, lipids	Improved FPG and lipid profile	Varies	<0.05
Nutritional Strategies for the Management of Type 2 Diabetes Mellitus: A Narrative Review [62].	Nutritional management	Review	HbA1c, insulin sensitivity	Not quantified	Not reported	Not reported
Nutrition in Patients with Type 2 Diabetes: Present Knowledge and Remaining Challenges [64].	Nutritional strategies	Review	Metabolic improvements	Not quantified	Not applicable	Not reported
Improving Type 2 Diabetes Mellitus Glycaemic Control Through Lifestyle Modification Implementing Diet Intervention: A Systematic Review and Meta-Analysis [65].	Diet intervention in T2DM	Meta-analysis	HbA1c reduction	-0.49	(-0.68, -0.30)	<0.001
Benefits of Continuous Positive Airway Pressure on Glycaemic Control and Insulin Resistance in Patients with Type 2 Diabetes and Obstructive Sleep Apnoea: A Meta-Analysis [76].	CPAP	202	HOMA-IR reduction	-1.05	(-1.91, -0.19)	0.016
Efficacy of Continuous Positive Airway Pressure (CPAP) Preventing Type 2 Diabetes Mellitus in Patients with Obstructive Sleep Apnea Hypopnea Syndrome (OSAHS) and Insulin Resistance: A Systematic Review and Meta-Analysis [77].	CPAP for OSA patients	Not specified	Improved HOMA-IR	-0.88	(-1.62, -0.14)	<0.05
Effects of a Ketogenic Diet in Overweight Women with Polycystic Ovary Syndrome [78].	Ketogenic diet (KEMEPHY)	14	HOMA-IR reduction	-0.53	(-0.67, -0.39)	<0.0001
Effects of a Vegetarian Diet Combined with Aerobic Exercise on Glycemic Control, Insulin Resistance, and Body Composition: A Systematic Review and Meta-Analysis [79].	Vegetarian + aerobic exercise	846	HOMA-IR reduction	-0.75	(-1.08, -0.42)	<0.001
Non-pharmacological Treatment Options in the Management of Diabetes Mellitus [80].	Non-pharmacological treatments	Review	Insulin sensitivity	Not quantified	Not reported	Not reported
Role of Insulin in Health and Disease: An Update [81].	Electroacupuncture + Metformin	Not specified	Increased insulin sensitivity	Significant improvement	Not reported	<0.05
Effect of Acupuncture and Metformin on Insulin Sensitivity in Women with Polycystic Ovary Syndrome and Insulin Resistance: A Three-Armed Randomized Controlled Trial [82].	Acupuncture *vs* Metformin	114	HOMA-IR reduction	-0.60	(0.10, 1.10)	<0.05
Insulin Resistance in Children [83].	Metformin in children	Not specified	Improved insulin sensitivity	Not quantified	Not reported	Not reported
Physical Activity is an Important Element in the Prevention and Treatment of Diabetes [85].	Aerobic + Resistance Exercise	Not specified	HbA1c reduction	-0.63	(-0.82, -0.45)	<0.01
Can Type 2 Diabetes Be Cured [86]?	Lifestyle + pharmacologic	Review	Glycaemic control	Conceptual support	N/A	N/A
Insulin Resistance: Impact on Therapeutic Developments in Diabetes [88]	Metformin mechanisms	Review	Insulin sensitivity	Conceptual support	N/A	N/A
Rapamycin/Metformin Co-Treatment Normalizes Insulin Sensitivity and Reduces Complications Of Metabolic Syndrome in Type 2 Diabetic Mice [92].	Metformin + Rapamycin	Not specified	Improved HOMA-IR	Improved over monotherapy	Not reported	<0.05
Insulin Resistance: From Mechanisms to Therapeutic Strategies [94]	Insulin resistance mechanisms	Review	Pathophysiology and therapy	Not quantified	Not reported	Not reported
Effects of Statin Therapy on Glycemic Control and Insulin Resistance: A Systematic Review and Meta-Analysis [100].	Statins and insulin resistance	Meta-analysis	Increased IR risk	Small but significant	Not reported	<0.05

## LIST OF ABBREVIATIONS

ADA: American Diabetes Association
DAG: Diacylglycerol
DM: Diabetes Mellitus
DPP-4: Dipeptidyl peptidase-4
EA: Electroacupuncture
FABP: Fatty Acid Binding Protein
GLP-1: Glucagon Like Peptide-1
HOMA-IR: Homeostasis Model Assessment of Insulin Resistance
LMIC: Low- and Middle-Income Countries
IDF: International Diabetes Federation
MNT: Medical Nutrition Therapy
NRCT: Non-Randomized Controlled Trials
OAD: Oral Antidiabetic Drug
OGTT: Oral Glucose Tolerance Test
RCT: Randomized Controlled Trials
SGLT: Sodium Glucose Cotransporter
TZDs: Thiazolidinediones
WHO: World Health Organization

## STANDARDS OF REPORTING

PRISMA guidelines and methodology were followed.

## SUPPLEMENTARY MATERIAL

PRISMA checklist is available as supplementary material on the publisher’s website along with the published article.
